# Grand Challenges in Biosensors and Biomolecular Electronics

**DOI:** 10.3389/fbioe.2021.707615

**Published:** 2021-08-06

**Authors:** Guozhen Liu

**Affiliations:** School of Life and Health Sciences, The Chinese University of Hong Kong, Shenzhen, China

**Keywords:** biosensors, biomolecular electronics, challenges, sensitivity, multiplex detection, continuous *in vivo* monitoring, device integration, sustainability

## Introduction

Biosensors are devices to provide the information on the presence or/and the concentration of an analyte in a biological or chemical reaction. Biomolecular electronics, instead, focus on the study of biological materials or biomolecules using electronic devices. Thus biosensors and biomolecular electronics (BBE) involve in the highly multi-disciplinary research within physics, chemistry, bioengineering, advanced materials, nanotechnology, biotechnology, and the clinical science. They are in the spotlight of biomedical sciences and healthcare. With the continuous threaten of the COVID-19 pandemic to global health, the role of BBE is becoming more and more essential in medical diagnostics and disease monitoring. Advances of technologies in the fields of chemistry (Kwok et al., 2015), biomaterials (Jiang et al., 2020), engineering (Qiao et al., 2020a), nanotechnology (Wang et al., 2018), biotechnology (Li et al., 2019a; Li et al., 2019b), have significantly boosted the development of this interdisciplinary forum BBE evidenced by the appearance of advanced sensing technologies in both academia and industry. The first biosensor was developed for oxygen detection in the 1950s. Since then, BBE has witnessed remarkable achievements in terms of sensing capability, sensing modality, sensing adaptability, sensing applications, et al. Being different from the traditional biosensing platforms on the bulk surface, the current biosensors are more versatile and compatible, such as disposable paper based biosensing devices (Dincer et al., 2019), printable biochips (Mikami et al., 2021), wearable biosensors (Sempionatto et al., 2019), implantable biosensors (Gray et al., 2018), ingestible biosensors Beardslee et al. (2020), and artificial intelligence assisted biosensing (Jin et al., 2020). These advanced biosensing platforms are paving the way to the era of digital health towards better health. Additionally, a biosensor can reach a sensitivity of a single cell level and even a single molecule level (Bhumkar et al., 2021). The commercially available nanopore technology for detecting a single molecule of DNA or RNA has made a significant breakthrough in BBE and played a pivotal role in battling the COVID-19 pandemic (Bull et al., 2020). A recent study shows a single molecule can be detected by a mobilephone microscope, which opens up a massive opportunity for point-of-care diagnostics with greatly enhanced sensitivity (Trofymchuk et al., 2021). SARS-CoV-2 infection can be screened by the traditional paper lateral flow assays with desirable sensitivity with the aid of the Clustered Regularly Interspaced Short Palindromic Repeats/Cas enzymes (CRISPR/Cas) technology (Broughton et al., 2020) and even in a wearable platform (Nguyen et al., 2021).

The increasing prevalence of chronic and lifestyle diseases (such as diabetes) boosts the applications of BBE in various industries. Besides the popular point-of-care devices such as oximetry, pregnancy test strips, and glucose test strips, continuous glucose monitoring systems are available in the market. BBE keeps on demonstrating its success in biomedical science and healthcare with its attractive features such as desirable sensitivity, high specificity, portability, end-user accessibility, fast result delivery, and adaptability to other technologies and devices. Because of the improved performance of biosensors and also the increasing demand for rapid point-of-care testing in a cost-effective fashion, the global biosensors market grows very fast (a compound annual growth rate (CAGR) of 7.9% from 2021 to 2028 is expected) (Liu et al., 2019; Liu et al., 2021). Despite the exciting future of BBE, the comprehensive clinical applications of BBE towards effective healthcare are on the way to be mature due to the below grand challenges.

## Sensitivity

It is extremely challenging to detect analytes (such as cytokines, protein post-translational modifications, circulating cancer cells) due to the high background signal in clinical samples (Liu et al., 2021). To enhance sensitivity, nanomaterials-based signal amplification is one of the most popular strategies by taking advantages of a big surface area to load the maximum number of biorecognition molecules (Liu et al., 2019). Nanozymes are the rising star in this aspect with benefits in terms of stability, cost, sensitivity and reaction speed although their specificity needs to be further improved (Liang and Yan, 2019). Being different from nucleic acid-based signal amplification extensively used for detection of nucleic acids or other analytes, CRISPR/Cas technology has made a breakthrough in the field of BBE since 2017 due to its extremely high sensitivity and specificity (Li et al., 2019a). A CRISPR/Cas12a autocatalysis-driven feedback amplification network enables the effective detection of genomic DNA in clinical samples with attomolar sensitivity (Shi et al., 2021). Besides sensitive assay development, in some cases, sampling is difficult, and sample volumes of body fluids (such as human breath, tears, cerebrospinal fluid) are small (Beale et al., 2017). Thus the joint efforts between sampling, assay development and device engineering are required to reach desirable detection. For example, cytokines are the trace amount of small proteins present in cerebrospinal fluid. The nature of cytokines under biological conditions can be affected by cytokine-binding proteins, inhibitors and soluble cytokine receptors (Liu et al., 2021). Interferences in the matrix of biological samples can cause false positive signals. Therefore, to make measurement accurate, methods or conditions related to sample collection and handling should be considered and specified. In order to achieve a reliable cytokine assay for *in situ* analysis in the brain or spinal cord, we developed a deployable device for sensitive detection of cytokines directly in brain space or spinal cord, which excluded the sampling process and could work *in vivo* (Zhang et al., 2018). Thus, besides the signal amplification strategies applied in bioassays, device engineering is also essential to increase sensitivity.

## Multiplex Capability

The multiplexing assays play a pivotal role in clinical practice towards precision diagnostics because they provide a comprehensive mapping of disease features and precise biological signature (Dincer et al., 2017). An assay capable of doing simultaneous detection of multiple analytes provides high-throughput sample information, requires less assay time and sample input, and reduces variations between singleplex assays. Challenges associated with the multiplex capability of BBE include limited signal readouts, cross-reaction, and sensitivity. Multiplex bead binding assays (such as Luminex multiplex assays) are the most popular multiplex assays in biomedical science and clinical. They are capable of doing the simultaneous analysis of multiple analytes in a clinical sample. How to make the assay to be cost-effective and instrument free is the driving force for developing the next generation of beads based multiplex analysis. It is challenging for electrochemical biosensors to achieve the simultaneous analysis of more than three analytes without signal overlap (Shen et al., 2021). Optical biosensors provide a better opportunity for multiple analysis by integrating with different optical tags such as SPR, SERS and upconverting nanoparticles (Pei et al., 2020). Additionally, advances in printing technologies powered bioassays with multiplex capability (Li et al., 2019b). Combining with advances in microfluidic technology and assay development, a lateral flow assay was able to detect seven pathogenic single nucleotide polymorphisms in a single test strip with the sensitivity of 0.04 pg ml^−1^. (Anfossi et al., 2019)^,^ (Liu et al., 2018) Although lots of opportunities for multiplexed analysis exist, few high-throughput platforms won the practical application in point-of-care detection, and the challenges lie in the lack of detection repeatability and device robustness.

## Continuous Monitoring *in vivo*

Effective healthcare relies on technologies for real-time monitoring of physiological conditions in order to continuously guard the state of health. While the modern biosensing technologies need to be powered with multiplexing capability, fast response, small sample volume, and high sensitivity, and achieving *in situ* real-time monitoring is another challenge associated with BBE. Advances in the fields of electronics and microfabrication techniques have significantly facilitated the interest in the use of wearable devices and implantable chips to realise the continuous monitoring of multiple health conditions (Yang and Gao, 2019). The trend for bioelectronics is to make the device biocompatible, flexible and multimodality. Over the past years, many wearable and implantable technologies are commercially available healthcare by sensing physical signals such as heart rate, muscle action, electrocardiography, etc. How to enable these sensors to simultaneously measure both physical properties and underlying biochemical processes is critically important for BBE.

A continuous real-time biosensing device is capable of providing the reversible signals of the analyte when the analyte concentration varies. And thus it can differentia signals between the analyte and the interferants in a complex sample environment (Cao et al., 2018). Meanwhile, it can directly provide the real-time signal of the analyte without additional operation steps. The existing challenge in the application of biosensors to continuous molecular monitoring is how to convert specific analyte information to measurable continuous output signals *in vivo* without background signal drift (Kang et al., 2017; Plaxco, 2020). This will require powering the detection device with functional units, for example, adding a component on the sensing interface with the antifouling capability to largely reduce nonspecific adsorption and thus enhance the signal-to-noise (Jiang et al., 2020). Meanwhile, radiometric measurement is helpful to eliminate the background drifting to realize the calibration-free biosensing devices for continuous molecular monitoring *in vivo* (Ni et al., 2020). Glucose continuous monitoring system is a symbolic example of BBE in achieving continuous molecular monitoring. The goal of reagentless, real-time biosensors for continuous monitoring of a spectrum of bioanalytes such as hormones, drugs, peptides, and proteins, that can be deployed directly in clinical samples providing real-time healthcare, remains largely unmet. In additional to realise continuous monitoring by chemistry, advances in wireless/Bluetooth signal readout are also essential to continuous monitoring *in vivo* (Qiao et al., 2020b). The interdisciplinary collaboration between fields of biomaterials, biosensors, bioelectronics, bioengineering, and the internet of things.

## Biosensing Device Integration and Commercialization

The current COVID-19 pandemic has significantly increased the demand for affordable and reliable laboratory diagnostics, especially in resource-limited countries. It is challenging to make these laboratory-developed biosensors to be qualified for commercial application as there is a huge gap between scientific knowledge and daily medical practice. Assay and device integration is essential to make them adaptable to real applications. Two hurdles exist for benchtop technologies walking out of the lab to the market (Burd, 2010): 1) the translation of laboratory-based technologies into clinical studies; 2) to translate clinical studies into medical practice. Current scientific research is focusing on passing the first hurdle as it can be managed through laboratory technology optimization. The second hurdle depends on the market effect since the new assays/devices need to be acceptable to the end-users/payers. Before entering the product market, any laboratory-developed assays or devices need to meet regulatory requirements, such as Food and Drug Administration (FDA), to ensure the accuracy, reliability, and appropriateness of clinical test results, regardless of where the test is performed (Genzen et al., 2017). Required performance characteristics of laboratory-developed technologies before implementation of FDA-approved/cleared tests include reportable range, analytical sensitivity, precision, analytical specificity, accuracy, and reference interval.

To cross the second hurdle, market specialty needs to be considered. For example, in the limited resources settings, in addition, to be accurate, robust, and easy to use, it requires the detection system to be affordable to end users. Current advances in paper-based microfluidic analytical devices, and mobile-phone based diagnosis have greatly contributed to this aspect. A disposable paper-based test strip can significantly low the detection cost and make it invasive by using a body fluid such as saliva or urine (Luo et al., 2020). By integration with personal equipment such as a mobile phone or a glucose test meter, self-quantitative detection can be performed by end-users without visiting the hospital. These simplified and integrated detection platforms are desirable for translating sensing technology into commercialization. But they face challenges (such as accuracy) Shrivastava et al. (2020), and require reliable assay/device standardization for these *in vitro* diagnostics (Lippi et al., 2016). Thus, extensive joint efforts between chemists, engineers, biologists, and clinicians, are necessary before clearing up the hazy road from bench to bedside for BBE.

## Sustainability to the Ecosystem

Disposable sensors are inexpensive and are in extremely high demand because nowadays people are well connected and expecting fast, accessible, and reliable information on our health conditions (Dincer et al., 2019). Meanwhile, the need for single-use sensors is crucial for avoiding contamination. While disposable biosensors bring us convenience and speed, they also cause concerns to our ecosystem. It requires introducing electronics to make biosensing devices smart. How to make our biosensors/biochips biodegradable is a big question to ask. Recent discoveries in the emerging space of sustainable sensing systems, including wearable devices, paper-based biochips, smartphone-based detection, recyclable biosensors, etc, have highlighted the necessity to develop a smarter, more user-friendly, cost-efficient, and environmentally friendly biosensing system. Researchers are dedicated to developing sustainable materials and sustainable systems for sensors. For example, distance-based biosensors on filter paper were developed for instrument-free semi-quantitative analysis (Cinti et al., 2019). Meanwhile, paper-based devices also feature as their low-cost, flexibale and capability of point-of-care testing with variable signal readout (Liu et al., 2019). Additionally, biodegradable silk or hydrogels has been widely applied in biosensing devices (Xu et al., 2019). It is challenging to achieve a sustainable sensing system with all desirable features, and a balance consideration needs to be reached for developing advanced and smart biosensing devices or bioelectronics with the desirable analytical performance which is also sustainable to the ecosystem (Singh et al., 2020).

In summary, modern BBE aims to realize precise diagnosis by developing devices/systems to achieve further advancement in terms of simplicity, sensitivity, specificity, affordability, multiplex capability, integration of different functions into a single biochip, *in vivo* sensing capability, and sustainability to the ecosystem. Bioelectronics have been focusing on the development of advanced materials and bio-signal processing technologies towards Intelligent of Things. Additionally, continuous development and validation of reliable biomarkers, development of new ultrasensitive transducer technology for profiling a range of biomarkers, detection automation, and verification/validation of the device in clinical testing are equally essential and challenging. It limits the rapid growth of these biosensing technologies to be clinically valuable in the market at a competitive cost. During the process to promote the cutting-edge research on creating smart, sensitive, and reliable biosensors and biomolecular electronics-ranging from handheld devices to implantable systems—to service better health, we expect the studies published in the specialty section of Biosensors and Biomolecular Electronics, under Frontiers in Bioengineering and Biotechnology, to contribute to solve our current challenges, while defining new challenges for the future towards big data and big health.
